# JODDD Editorial policy on artificial intelligence: Editorial workflow, author and reviewer responsibilities, and ethical considerations

**DOI:** 10.34172/joddd.025.44233

**Published:** 2025-09-30

**Authors:** Naser Asl Aminabadi

**Affiliations:** Department of Pediatric Dentistry, Faculty of Dentistry, Tabriz University of Medical Sciences, Tabriz, Iran

## Introduction

 The rapid emergence of artificial intelligence (AI) has transformed multiple facets of scholarly publishing, including manuscript preparation, peer review, and editorial management. From plagiarism detection and reference validation to image analysis, language support, and reviewer selection, AI offers unprecedented opportunities to enhance efficiency, inclusivity, and overall quality assurance. However, its integration into the editorial workflow also presents ethical, methodological, and practical challenges.

 As a journal committed to transparency, equity, and academic integrity, the *Journal of Dental Research, Dental Clinics, Dental Prospects* (JODDD) recognizes that while AI can strengthen editorial processes and improve clarity for authors, its use must always remain under human oversight. These technologies must be applied responsibly—without compromising originality, fairness, confidentiality, or the irreplaceable role of human judgment.

 Accordingly, this policy provides clear guidance for authors, reviewers, and editors on the responsible use of AI in manuscript preparation, peer review, and editorial workflow. By aligning with international standards and reinforcing human oversight, JODDD reaffirms its mission to advance dental science with fairness, accountability, and integrity.

 In keeping with this commitment and in alignment with global best practices, JODDD formally introduces this editorial policy on the use of AI in scholarly publishing.

## Opportunities and Challenges of AI

 When applied appropriately, AI can support and enhance editorial practice by:

- Streamlining initial triage through similarity checks, reference formatting, and image duplication detection
- Improving clarity and readability, particularly for authors writing in English as a second language
- Assisting in reviewer identification through analysis of scholarly networks and publication records
- Detecting errors such as statistical inconsistencies, missing ethical statements, or duplicated content

Nevertheless, reliance on AI also introduces challenges:

- Bias and inequity: Algorithms trained on limited datasets may disadvantage submissions from underrepresented regions or minority populations.
- Lack of contextual judgment: AI cannot replace human evaluation of scientific originality, contextual relevance, or ethical nuance.
- Risk of overdependence: Excessive reliance may diminish essential human oversight in editorial decision-making.
- Confidentiality concerns: Uploading manuscripts to unsecured AI platforms may compromise author privacy and intellectual property.
- Transparency gaps: Many AI systems function as ‘black boxes,’ limiting accountability, reproducibility, and trust.

## Responsibilities of Authors, Reviewers, And Editors

###  Authors

- Disclosure: Authors must declare any use of AI (e.g., text editing, figure generation, or data analysis) in the Acknowledgments or Methods section.
- Accountability: Authors remain fully responsible for the accuracy, originality, and integrity of all manuscript content.
- Authorship: AI tools cannot be listed as authors, as authorship requires human accountability and intellectual contribution.

###  Reviewers

- AI assistance: Reviewers employing AI (e.g., for grammar checks or summarization) must disclose this in their confidential report to editors.
- Confidentiality: Manuscripts must never be uploaded to unsecured or public AI platforms.

###  Editors

- Support, not substitute: While JODDD uses similarity-detection tools such as iThenticate, editorial decisions remain the responsibility of human editors.
- Bias oversight: Editors must remain vigilant for biases or errors introduced by AI systems.
- Transparency: The journal will clearly communicate any use of AI in its editorial and review processes.

###  Ethical Principles

- Human oversight remains paramount and irreplaceable.
- Equity and fairness must guide all editorial decisions.
- Transparency and accountability are essential to maintaining trust.
- This policy aligns with guidance from the Committee on Publication Ethics (COPE)^1^, the International Committee of Medical Journal Editors (ICMJE)^2^, the World Association of Medical Editors (WAME)^3^, and JODDD’s editorial framework.

## Policy Review

 Given the rapid evolution of AI technologies^4^, this policy will be reviewed annually. Updates will be published on the JODDD website and communicated in future editorials.

## Competing Interests

 The author is the Editor-in-Chief of the Journal of Dental Research Dental Clinics Dental Prospects (JODDD). The author declares no other competing interests concerning authorship and/or publication of this article.

## Ethical Approval

 Not applicable.
